# Promotion of the occurrence of endometrioid carcinoma by S100 calcium binding protein P

**DOI:** 10.1186/s12885-020-07350-x

**Published:** 2020-09-03

**Authors:** Dan Zhang, Xiuying Chen, Hexia Xia, Lu Wang, Hongbo Zhao, Bufang Xu, Aijun Zhang, Wei Zhang

**Affiliations:** 1 Obstetrics and Gynecology Hospital, Fudan University, Postal address: 413 Zhaozhou Road, Shanghai, 200011 China; 2 Reproductive Medical Center of Ruijin Hospital, School of Medicine, Shanghai Jiao Tong University, 197 Ruijin Second Road, Shanghai, 200025 China

**Keywords:** S100 calcium binding protein P, Endometrial cancer, Ezrin, Cell invasion, Cytoskeletal rearrangement

## Abstract

**Background:**

Endometrial cancer, one of the most common malignant tumors, is a serious threat to women’s health. Endometrial hyperplasia is a precursor of endometrial cancer. S100 calcium binding protein P (S100P) has been found to play important roles in many types of cancer. The present study aimed to investigate the expression of S100P in endometrial cancer and its precursor lesions, and to explore the possible mechanisms.

**Methods:**

We collected paraffin sections of normal endometrium, simple and complex non-atypical hyperplasia, atypical hyperplasia, and endometrioid carcinoma. The expression of S100P in endometrial cancer and its precancerous lesions was observed using immunohistochemistry. We also cultured primary endometrial cells and endometrial cancer cell lines (Ishikawa and RL95–2), and observed the expression of S100P in these cells. Laser confocal microscopy was used to observe the co-localization of S100P and its interacting protein Ezrin in RL95–2 cells. We employed lentiviruses to knockdown and overexpress *S100P* and then detected the F-actin distribution and cell invasion using phalloidin staining and Transwell assays.

**Results:**

There was a gradual increase in the S100P signal as the disease progressed from normal endometrium and simple non-atypical hyperplasia, to complex non-atypical hyperplasia, atypical hyperplasia, and then to endometrial cancer. S100P was mainly distributed in the cytoplasm and co-localized with Ezrin in endometrial cancer cells. After knocking down *S100P*, F-actin aggregated in the nucleus or to the local cell membrane. Furthermore, knockdown of *S100P* in Ishikawa cells decreased their cell invasion capability. Meanwhile, *S100P* overexpression in endometrial stromal cells increased cell invasion.

**Conclusions:**

These data suggested that S100P might be involved in the occurrence and development of endometrial cancer via interaction with Ezrin and re-organization of F-actin to promote cell invasion.

## Background

Globally, there are more than 380,000 new cases of endometrial cancer each year, which seriously threatens women’s health [1, 2]. In China, the incidence of endometrial cancer is also gradually increasing, with approximately 63,400 new cases diagnosed in 2015 [3]. Endometrial cancer (EC) has two recognized clinical subtypes: type I (estrogen-dependent, endometrioid carcinoma) and type II (non-estrogen-dependent, non-endometrioid carcinoma) [4]. Between them, type I accounts for more than 80% of the total number of cases [5].

Endometrial hyperplasia (EH) represents a series of morphological endometrial pathological changes that are at risk of developing into EC [6]. The WHO 1994 classifications of EH categorized it as simple or complex hyperplasia, with or without atypia. The new WHO 2014 classification categorized EH as non-atypical EH and atypical EH [7]. Although EH with cytological atypia is characterized as a direct precancerous lesion of EC, simple hyperplasia shows a low risk of cancer progression, and complex hyperplasia has an intermediate risk of progression [6].

The incidence of EC is related to excessive exposure of the uterus to estrogens and the imbalance between estrogens and progesterone, which involves a series of molecules [5]. S100 calcium binding protein P (S100P) is a member of the S100 small molecular weight (9–14 kDa) calcium-binding protein family [8]. It is functionally implicated in many cancers, such as cholangiocarcinoma, breast cancer, and pancreatic ductal adenocarcinoma [9–11]. It participates in the regulation of proliferation, invasion, survival, metastasis, angiogenesis, and resistance to chemotherapy drugs to promote the occurrence and development of cancer [12–14].

In our previous studies, we found that S100P is expressed in normal endometrial cells and is upregulated in the endometrium at the mid-secretory phase, participating in the formation of endometrial receptivity [15, 16]. Recent studies suggested that S100P functionally improves the proliferation and invasion of EC [17, 18]. However, whether S100P is involved in the continuous progress from precursor lesions to type 1 EC, and its related mechanism, is unclear. In the present study, we investigated the expression of S100P in type 1 EC and EH, and demonstrated the effects of S100P on cell invasion and cytoskeletal remodeling.

## Methods

### Sample collection

Paraffin-embedded sections were collected from the tissue storage unit of the Obstetrics and Gynecology Hospital of Fudan University. The inclusion criteria included: Age 20 to 45 years old with EH or EC histologically confirmed by the Pathology Department of the Obstetrics and Gynecology Hospital of Fudan University. The exclusion criteria included patients that received hormonal therapy or chemotherapy within three months before sample collection. A total of 21 samples were collected. Among them, five samples were diagnosed as normal endometrium of proliferative phase, three were SH, five were CH, three were AH, and five were grade-1 type 1 EC.

Benign proliferative endometrium samples for cellular tests were obtained via curettage after hysteroscope or using a pipette catheter. The inclusion criteria included: Age between 20 to 40 years old, and with regular menstrual cycles (27–33 d). The exclusion criteria included: Patients with endometriosis, or with any other endometrial lesions, such as endometrial hyperplasia and polyps and intrauterine adhesions; patients with an intrauterine device or who received steroid hormone medication within 3 months.

### Primary cells and cell line culture

Primary endometrial epithelial cells and endometrial stromal cells (EECs and ESCs) were separated according to a previously described method [19]. The dispersed endometrial cells were separated into ESCs and EECs by filtering through a 40-μm pore size nylon cell strainer. The purity of stromal and epithelial cells was determined using vimentin and cytokeratin cell staining and both were > 95%.

The human uterine endometrial cancer RL95–2 cell line (CRL-1671) was purchased from the American Type Culture Collection (ATCC, Manassas, VA, USA). The Ishikawa endometrial cancer cell line was a gift from the staff of Fudan University-affiliated Gynecology and Obstetrics Hospital. The cells were maintained in DMEM/F12 (Gibco) containing 10% fetal bovine serum (FBS), at 37 °C in a humid atmosphere with 5% CO_2_.

### RNA isolation and real-time PCR analysis

We used the Trizol reagent (Invitrogen, Waltham, Massachusetts, USA) to isolate total RNA and used RevertAid™ First Strand cDNA Synthesis Kit (Fermentas, Burlington, Canada) to generate cDNA according to the manufacturer’s protocol. Quantitative PCR reactions were performed using SYBR Premix Ex Taq (Takara, Shiga, Japan) and detection was performed using the Applied Biosystem PRISM 7500HT system (Applied Biosystems, Foster City, CA, USA). In all samples, *ACTB* (encoding β-actin) was used as an internal reference to assess the expression of *S100P*. We use the online software Primer 3 to design primers at http://frodo.wi.mit.edu/, as shown in Table 1. All experiments were performed in triplicate.

**Table 1:** Sequences of primers used in the present study

Primer	Forward	Reverse
S100P	5′- TACCAGGCTTCCTGCAGAGT − 3′	5′-AGGGCATCATTTGAGTCCTG-3′
ACTB	5′-CGGGACCTGACTGACTACTCA-3′	5′-TCAAGAAAGGGTGTAACGCAACTA-3′
S100P-O^a^	5′-CGGAATTCTGGGTCTGAATCTAGCACCATGACG-3′	5′-CGGGATCCAGGGCATCATTTGAGTCCTGCCT-3′
S100Pi-1^b^	5′-CCGGAAGGATGCCGTGGATAAATTGCTCGAGCAATTTATCCACGGCATCCTTTTTTTG-3′	
S100Pi-2 ^b^	5′-CCGGTGCCGTGGATAAATTGCTCAACTCGAGTTGAGCAATTTATCCACGGCATTTTTG-3′	
S100Pi-3 ^b^	5′-CCGGCTGTCACAAGTACTTTGAGAACTCGAGTTCTCAAAGTACTTGTGACAGTTTTTG-3′	
Scrambled ^c^	5′-CCGGCCTAAGGTTAAGTCGCCCTCGCTCGAGCGAGGGCGACTTAACCTTAGGTTTTTG-3′	

^a^ Primers for S100P overexpression

^b^ Sequences used for S100P interference

^c^ Sequences of the Scrambled control

### Cellular immunofluorescence staining

Cell monolayers cultured in 96-well dishes or on coverslips were fixed with 4% paraformaldehyde. After permeabilization with 0.1% Triton X-100 for 45 min, cells were incubated with 1:50 mouse anti-human S100P monoclonal antibody (R&D Systems, Minneapolis, MN, USA) as the primary antibody, and then incubated with 1:500 Cy3-labeled goat anti-mouse IgG (Invitrogen) as the secondary antibody. Alternatively, the cells were stained with 5 μg/ml FITC-phalloidin (Sigma) for F-actin staining. Cell nuclei were counterstained with Hoechst33258 stain (Sigma) at 1 mg/ml. The cells were then observed under a fluorescence microscope (Axiovert 200, Zeiss, Jena, Germany).

### Tissue Immunohistochemical staining

Paraffin-embedded sections were subjected to a two-step immunohistochemical approach. Formalin-fixed and paraffin embedded sections (4 μm) were deparaffinized, hydrated, and heated in an oven for antigen retrieval, before being covered with 0.3% (w/v) hydrogen peroxidase for 30 min to inactivate the endogenous peroxidase. A rabbit monoclonal antibody against S100P (1:150, ab124743, Abcam Cambridge, MA, USA) was used as the primary antibody. Phosphate-buffered saline (PBS) instead of primary antibody was used as the negative control.

### Cell processing for laser scanning confocal microscopy

RL952 cells were seeded at 1 × 10^5^ per well into a 24-well plate in which coverslips had been preset into the wells. The cells were fixed in 4% paraformaldehyde after 24 h of culture to achieve a aggregation rate of about 50%. After permeabilization with 0.1% Triton X-100 for 45 min, rabbit anti-human Ezrin 1: 50 dilution (Epitomics, Burlingame, CA, USA; EP886Y) and mouse anti-human S100P 1:50 dilution (R&D Systems) were used as the primary antibodies. DyLight 680 labeled Goat anti rabbit IgG 1:500 dilution (KPL, Gaithersburg, MD, USA) and DyLight 800-labeled Goat anti mouse IgG 1: 500 dilution (KPL) were used as the secondary antibodies. Hochest33258 solution (diluted with 5% fetal bovine serum PBS) at a concentration of 1:1000 was added for nuclei staining. The cells were viewed and scanned under a Leica Confocal scanning microscopy (Wetzlar, Germany) after a fluorescent sealing tablet (Antifade mounting medium; Beyotime, Jiangsu, China) was added.

### Lentiviral vectors construction and infection

The stuffer DNA was removed from plasmid pLKO.1 by AgeI/EcoRI digestion and replaced with double stranded oligonucleotides encoding the desired shRNA. The target-specific shRNA sequences used in this study were designed using tools from Sigma (TRC, MISSION®TRC shRNA library, Sigma) and the target sequences are shown in Table 1. The *S100P* cDNA was amplified together with EcoRI/BamHI restriction sites and sub-cloned into plasmid pCDH-CMV-MCS-EF1-copPuro to generate overexpression plasmids. The respective primer sequences are listed in Table 1. Lentiviruses were generated by co-transfecting 293 T cells with 4 μg of shRNA-encoding plasmid, 3 μg of psPAX2, and 1 μg of pMD2.G plasmids using Lipofectamine2000 (Invitrogen). Growth media was exchanged the following day and supernatants were collected every 12 h during two consecutive days after 48 h of transfection. Target cell lines were infected with the constructed lentiviruses in the presence of polybrene (6 μg/ml) and selected in 2 μg/ml puromycin two days later. The efficiency of interference and overexpression was tested by immunofluorescence staining, and the construct with the highest interfering efficiency of the three target sequences (S100P interference-1) was used for the follow-up study. The raw data of *S100P* overexpression and knocked-down plasmid sequencing were uploaded as supplementary data.

### Cell invasion assay

Invasion of cells through 8-μm pores were assessed using a Transwell® Cell Culture chamber (Corning Costar, NY, USA) coated with Matrigel™ (BD Pharmingen, San Diego, CA, USA). DMEM/F12 medium (600 μl) supplemented with 15% fetal bovine serum (FBS) was added to the lower chamber and 1 × 10^4^ cells in 100 μl of serum-free medium were added to the upper chamber and incubated for 2 h at 37 °C. After removing the non-invading cells from the upper surface of the membrane, the cells that moved through the pores were fixed in 4% paraformaldehyde and stained with 1% Giemsa blue (Sigma-Aldrich). The membranes were photographed and cells were counted in the central field of triplicate membranes.

### Statistical analysis

Densitometric analysis was conducted to compare the expression level of proteins using Image J (Version 1.5.1; NIH, Bethesda, MD, USA). The results were analyzed statistically using SPSS (Version 23, IBM Corp., Armonk, NY, USA). Results are presented as mean ± SD of three to six independent experiments. A T-test or one-way analysis of variance (ANOVA), followed by Tukey’s multiple comparisons post hoc test, were performed to analyze statistical significance. * *p* < 0.05 indicated a statistically significant difference.

## Results

### S100P is highly expressed in endometrial cancer cells

**Fig. 1:**
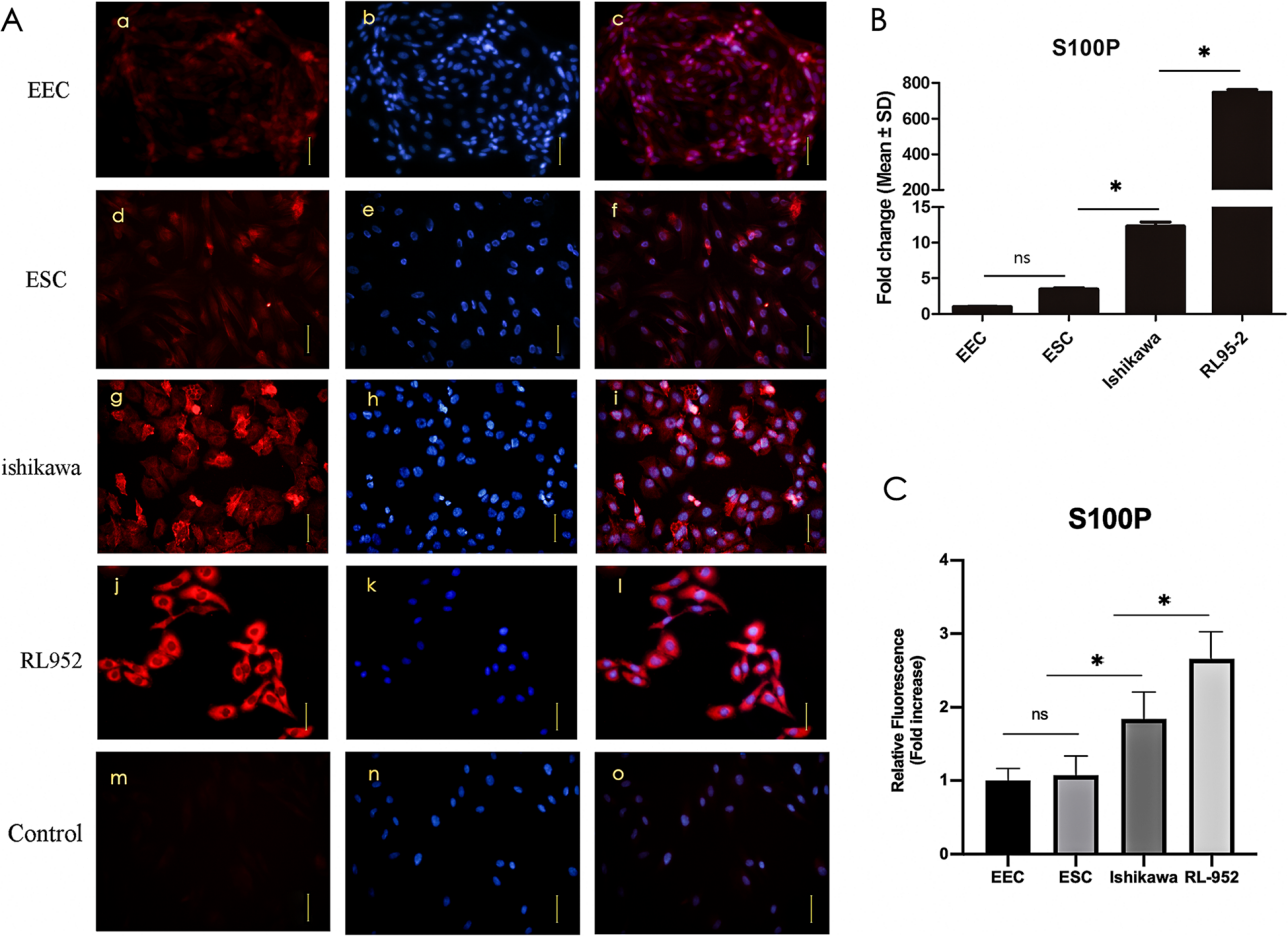
Expression of S100P in endometrial cells. A: Immunofluorescence staining for S100P (red) and Hoechst33258 (blue) in EECs (a, b, c), ESCs (d, e, f), Ishikawa cells (g, h, i), and RL95–2 cells (j, k, l). PBS was used as a negative control (m, n, o), and the scale bar was 50 μm). B: The mRNA expression of *S100P* in different endometrial cells, as determined using qPCR. C: Fluorescence densitometric analysis using image J, normalized to that of EECs. EEC: primary endometrial epithelial cells, ESC: primary endometrial stromal cells, * *P* < 0.05

The results of real-time PCR analysis showed that the *S100P* mRNA was highly expressed in RL95–2 and Ishikawa cells. Compared with primary endometrial epithelial cells, its expression was approximately 15-fold higher in Ishikawa cells and nearly 800-fold higher in RL95–2 cells (*p* ≤ 0.01) (Fig. 1B). The results of immunofluorescence showed that the S100P protein had a similar expression trend to the mRNA in endometrial cancer cells. The fluorescence signal intensities of S100P in these two cancer cell lines were significantly stronger than those of primary endometrial epithelial cells and stromal cells. Furthermore, the fluorescence signal in RL95–2 cells was much stronger than that in Ishikawa cells, and the difference was statistically significant. Fluorescent signals were distributed in the cytoplasm and nucleus, but mainly in the cytoplasm (Fig. 1A and C).

### S100P gradually increases from complex, atypical hyperplasia, and to endometrial cancer

**Fig. 2:**
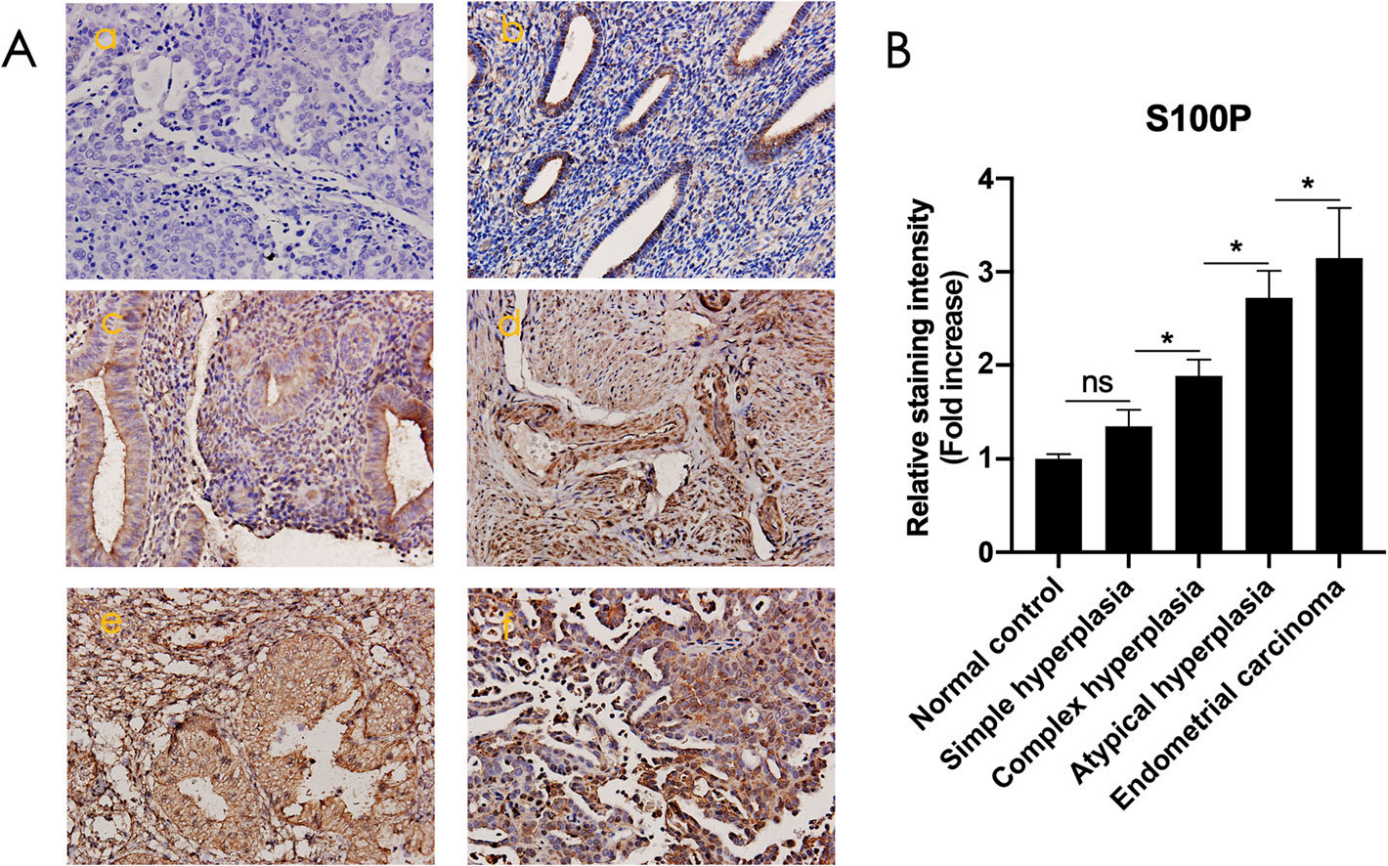
Expression of S100P in endometrial cancer and its precursor lesions. A: Immunohistochemistry demonstrating the distribution of S100P in endometrial cancer and its precursor lesions. a, Negative control; b, Normal endometrium in the proliferative phase; c, Endometrium of SH; d, Endometrium of CH; e, Endometrium of AH; f, Endometrium of EC (Magnified 200×). B: Densitometric analysis using image J. The data were normalized to the normal control. ns: no significance; * *P* < 0.05

Immunohistochemical staining was performed to detect the expression and distribution of S100P in the tissue samples of endometrial cancer and its precursor lesions. The results showed that as the disease progressed, the S100P signals gradually increased. There was no significant difference between SH and the normal endometrial control; however, as the disease progressed to CH, AH, and EC, a significant increase in S100P levels was observed between the groups. The enhanced S100P signal was distributed in the stroma, glandular and lumen epithelium, and accumulated mainly in the glandular epithelium (Fig. 2).

### S100P improves the invasion of endometrial cells

**Fig. 3:**
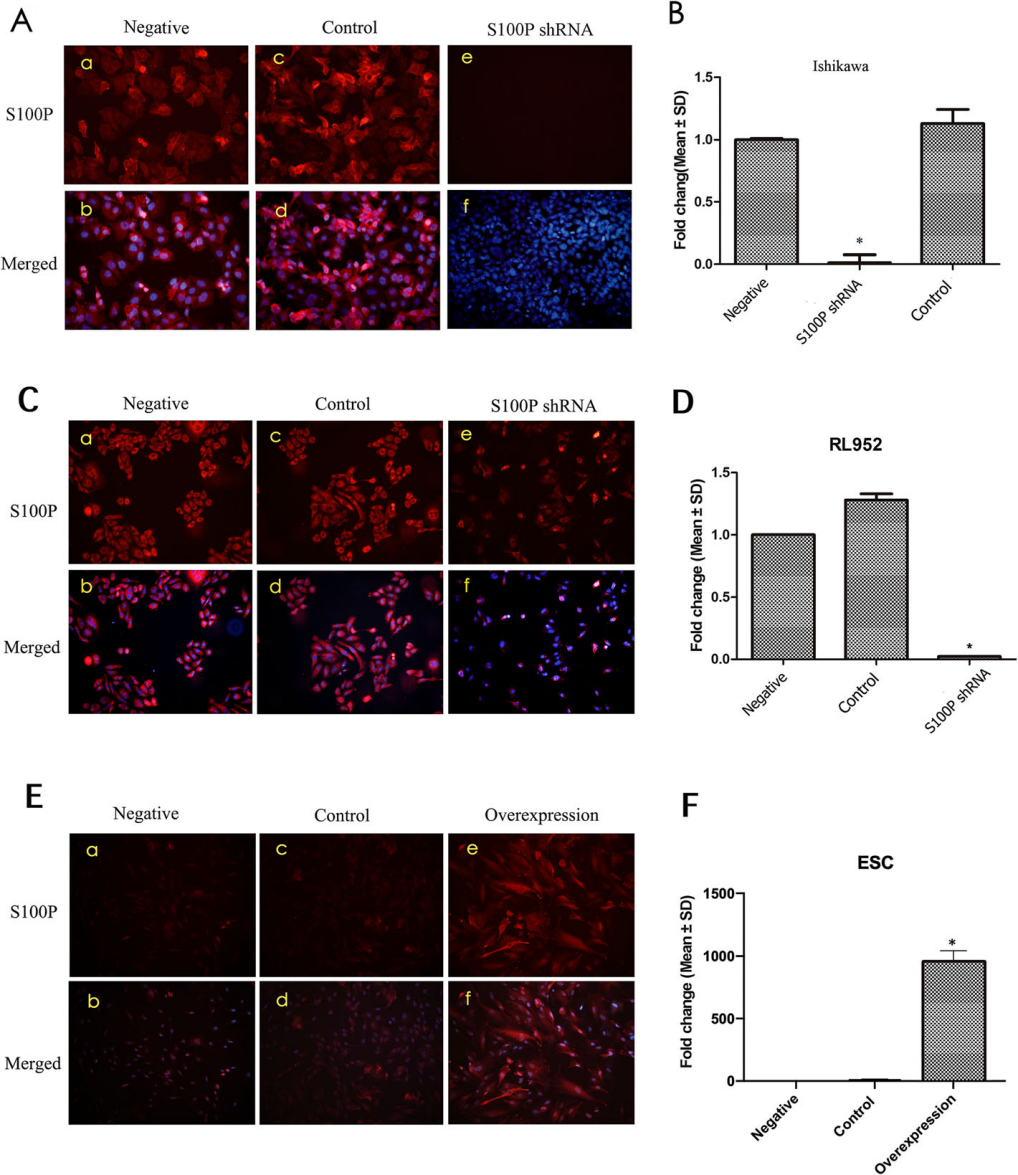
The efficiency of *S100P* knockdown and overexpression in cells. A, C: Knockdown of *S100P* (red) in Ishikawa or RL95–2 cells, respectively; Hoechst33258 (blue) for nuclear staining (Magnified 200 times). B, D: The changes in the *S100P* mRNA level in A or C, as determined using qPCR. *ACTB* (β-actin) was used as the internal reference (* *P* < 0.05). E, F: overexpression of *S100P* (red) in primary endometrial stromal cells and changes in the *S100P* mRNA level as mentioned above. Negative: uninfected cells; S100P shRNA: cells infected with *S100P* interference lentiviruses; *S100P* overexpression: cells infected with *S100P* overexpression lentiviruses; Control: Cells infected with scrambled sequence lentiviruses (A, B, C, D) or empty vector (E, F)

The expression of S100P was inhibited by more than 95% at both the mRNA and protein levels after infection with the S100P shRNA lentiviruses, and the cells infected with scrambled sequence showed no significant change in S100P levels (Fig. 3A, B, C and D). We then overexpressed *S100P* in primary endometrial stromal cells. The results showed that the S100P level increased markedly in the transfected cells (Fig. 3E and F).

**Fig. 4:**
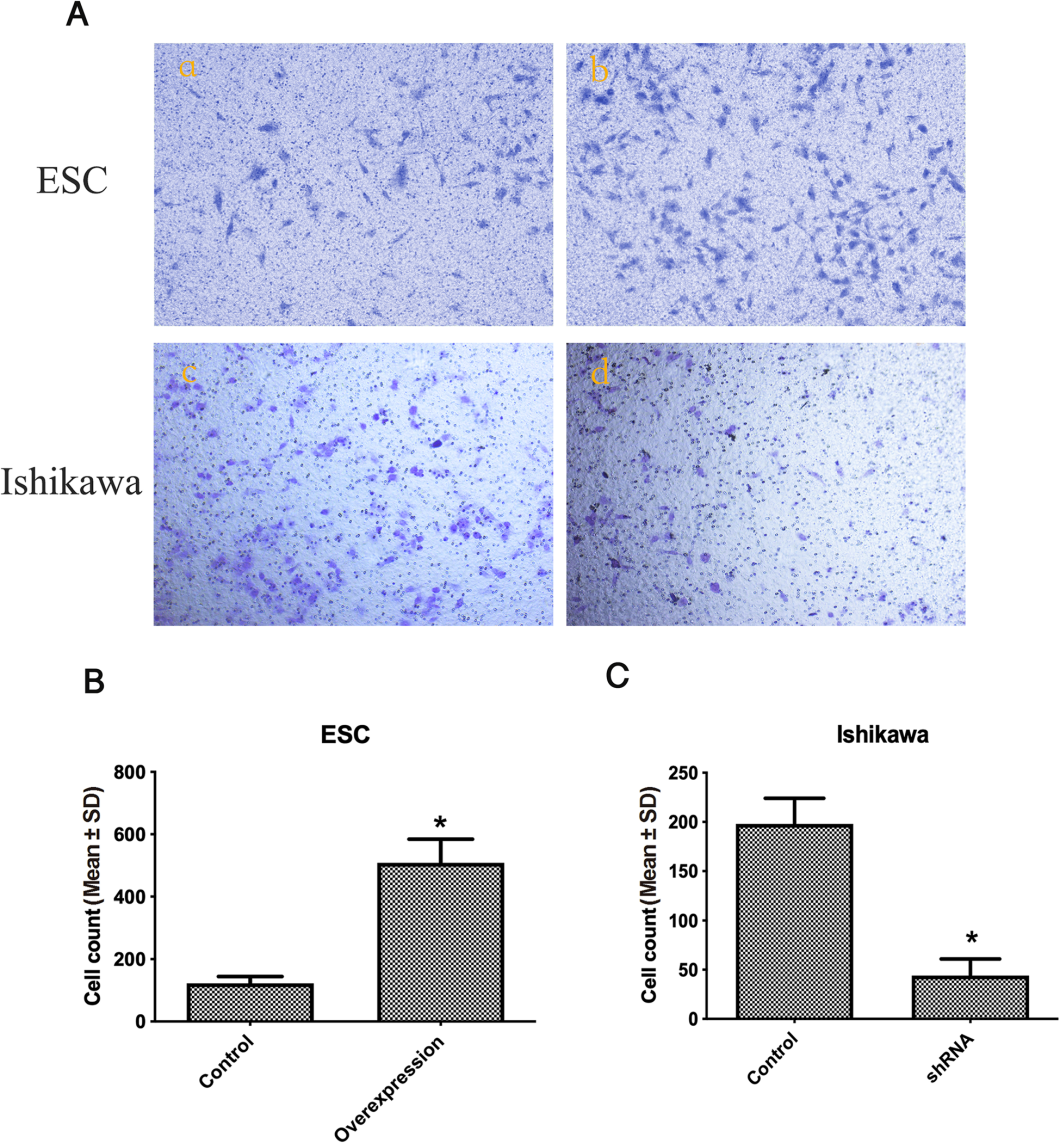
Transwell experiments showing the effects of S100P on cell invasion. A: The invasive capacity of Ishikawa cells or endometrial stromal cells after silencing or overexpression of *S100P* using lentiviruses was determined using Transwell assays. Representative images (magnified 200 times) were captured at 48 h after the cells were seeded. All the experiments were performed in triplicate. a, b: ESCs transfected with the empty vector or showing S100P overexpression, respectively; c, d: Ishikawa cells with the scramble vector or subjected to *S100P* downregulation, respectively. B and C: Histogram showing the number of invasive cells per field from three independent experiments. *P < 0.05

The results of cell invasion assays showed that the number of invading cells decreased significantly after *S100P* silencing in Ishikawa cells, while the number of invading cell increased after *S100P* overexpression in ESC cells (Fig. 4).

### S100P co-locates with EZRIN and causes F-actin rearrangement

**Fig. 5:**
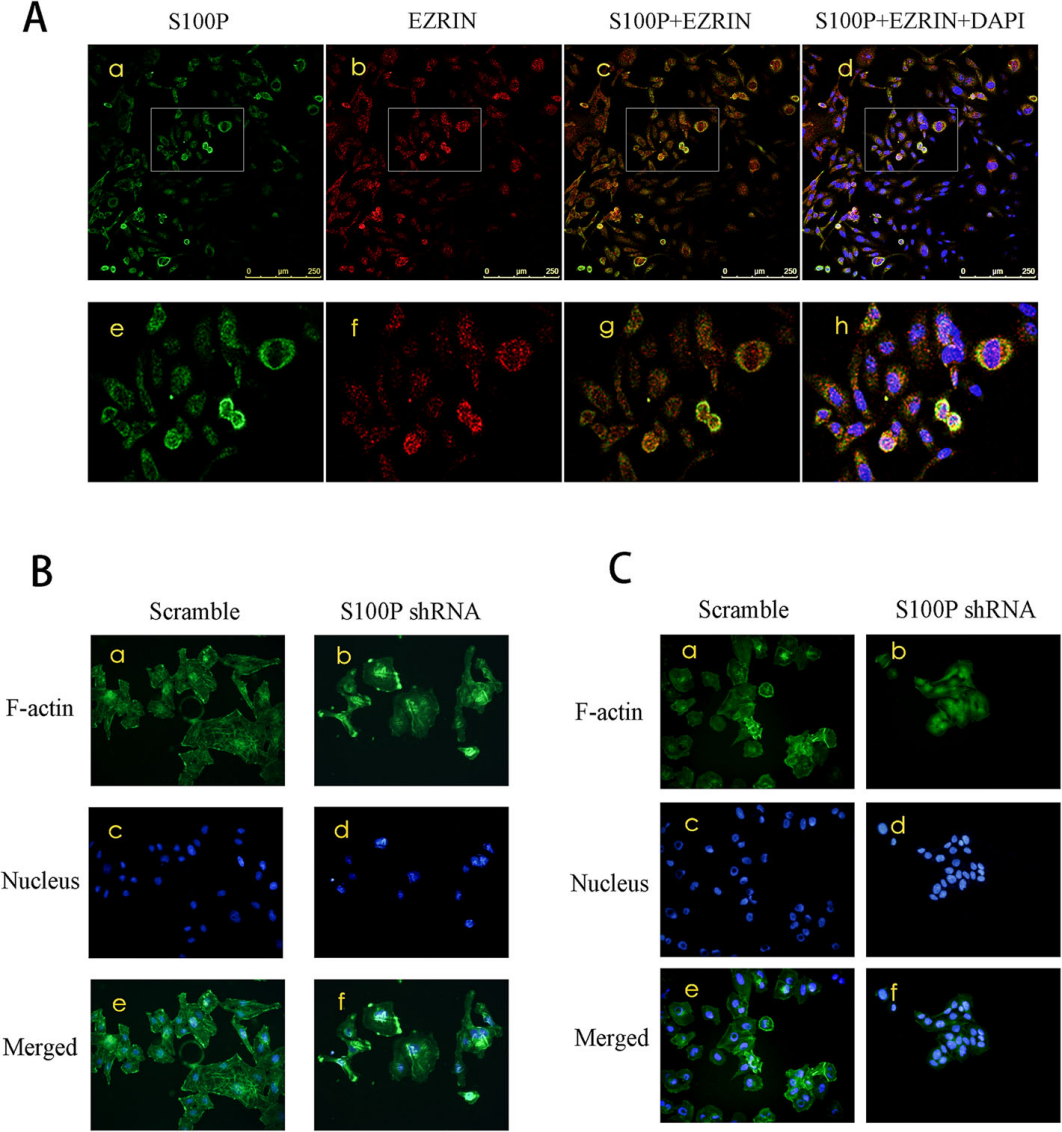
The colocalization of S100P and Ezrin, and F-actin rearrangement after knocking down *S100P*. A: Laser confocal microscopy showing the colocalization (yellow) of S100P (green) and Ezrin (red) in RL95–2 cells. The panels below (e, f, g, h) are an enlargement of the box area (a, b, c, d). B and C: FITC-phalloidin (green) staining showing the rearrangement of F-actin in RL95–2 cells (left) and Ishikawa cells (right). Scramble control (a, c, e) or *S100P* shRNA (b, d, f) set as mentioned previously. The nuclei were counterstained using Hoechst33258 (blue). The magnification of (A) is displayed using a ruler and the magnification in B and C was 400 ×

We used laser confocal microscopy to detect the colocalization of S100P and EZRIN in RL95–2 cells. The results suggested that the distribution of S100P and EZRIN in cells was consistent and co-localized (Fig. 5a). Phalloidin staining showed that F-actin fibers were evenly arranged in RL95–2 and Ishikawa cells. Knocking down *S100P* in both RL95–2 and Ishikawa cells caused F-actin fiber reorganization, in which they tended to aggregate to the nucleus or local membrane (Fig. 5B and C).

## Discussion

In the present study, we revealed that S100P is involved in the progression of endometrial cells from precancerous lesions to endometrioid carcinoma. The significantly higher expression of S100P in endometrial cancer cells suggested that it might play important roles in endometrial cancer, as it does in other cancers [20, 21]. Furthermore, RL95–2 cells are derived from a grade 2 moderately differentiated adenosquamous carcinoma, while Ishikawa cells originate from a well differentiated human endometrial adenocarcinoma [22]. Therefore, the much higher expression of S100P in RL95–2 cells than in Ishikawa cells indicated that the expression of S100P might be related to the degree of malignancy of endometrial cancer. A recent article reported that the expression of S100P progressively increased from grade 1 to grade 3 endometrial cancers, which supported our hypothesis [17].

Moreover, the observation that S100P signals increased in CH and AH, indicated that S100P is expressed early in endometrial tumorigenesis and might play a positive role in the development and progression of endometrioid carcinoma. This contrasted with the opinion of Jiang, et al., who discovered that S100P was highly expressed in endometrial squamous cell and adenosquamous carcinomas, but not in adenocarcinoma [18]. These differences might be caused by the sensitivity of the experiments. In our study, the expression of S100P in adenosquamous carcinoma-derived RL95–2 cells was much higher than that in adenocarcinoma-derived Ishikawa cells, which supported Jiang’s opinion that S100P is overexpressed in endometrial adenosquamous carcinomas. However, the expression of S100P in adenocarcinoma-derived Ishikawa cells was also much higher than that in normal endometrial epithelial and stromal cells. Another study reported that the expression of S100P was elevated in endometrial cancer [17]. In that study, all the histological subtypes of samples examined were endometrioid, which supported our results. Regrettably, because of difficulties in sample collection, our sample size is small and we were unable to collect fresh samples from different endometrial hyperplasia for western blotting and real-time PCR verification. Further studies with expanded sample sizes are required to form more reliable conclusion.

Previous studies showed that S100P was localized in the cytoplasm and/or nucleus of a wide range of cells, and could also be secreted to extracellular regions [23–25]. Our results showed that highly expressed S100P mainly accumulated in the cytoplasm and was weakly detected in the nucleus in Ishikawa and RL95–2 cells. It has been reported that the intracellular component of S100P could interact with the cytoskeletal protein Ezrin, which plays pivotal roles in the rearrangement of the cytoskeleton and participates in cell movement [26, 27]. Our laser confocal microscopy results showed the co-localized expression of S100P and EZRIN in endometrial cancer cells, which indicated that S100P might interact with Ezrin during the occurrence and progress of endometrial cancer. Interestingly, increased levels of Ezrin have also been reported in atypical endometrial hyperplasia and uterine endometrioid adenocarcinoma [28–30]. These reports further supported our hypothesis.

Ezrin is a principal member of the ERM (ezrin–radixin–moesin) protein family, which are known as structural organizers that link membrane proteins to the underlying actin cytoskeleton [31]. Phalloidin staining demonstrated obvious reorganization of F-actin in both RL95–2 and Ishikawa cells after knockdown of *S100P*. This indicated that S100P might interact with Ezrin to influence the organization of F-actin in endometrial cancer cells. Rearrangement of F-actin fibers is crucial to the formation of membrane protrusions at the leading-edge during cell movement [32]. The results of the present study revealed that knockdown of *S100P* in both RL95–2 and Ishikawa cells caused F-actin to aggregate to the nucleus or local membrane, which might impede cell movement. The invasion assay confirmed that downregulation of *S100P* inhibited cell invasion, while overexpression of *S100P* enhanced it. Thus, S100P is likely to promote the invasion and progression of endometrial cancer.

### Uniqueness and limitation

The uniqueness of this study is that we observed the S100P signal in EH and displayed the distribution of F-actin after *S100P* knockdown. However, the small sample size was our biggest limitation, and further expansion of the sample size is needed in future studies.

## Conclusions

In summary, S100P is expressed highly in endometrial cancer cells, and increases gradually from normal endometrium and SH to CH, to AH and then to EC. S100P influences cell invasion by interacting with Ezrin and reconstructing F-actin, which implies its involvement in the occurrence and development of endometrial cancer.

## Supplementary information

**Additional file 1.**

## Data Availability

The raw data of *S100P* overexpression and knocked-down plasmid sequencing were uploaded as supplementary data. The other datasets used and/or analyzed during the current study are available from the corresponding author on reasonable request.

## Abbreviations

S100P: S100 calcium binding protein P
EH: endometrial hyperplasia
EC: endometrial cancer
SH: simple non-atypical hyperplasia
CH: complex non-atypical hyperplasia
AH: atypical hyperplasia
EECs: endometrial epithelial cells
ESCs: endometrial stromal cells
DMEM: Dulbecco's Modified Eagle Medium
BSA: bovine serum albumin
PBS: phosphate-buffered saline
ERM: ezrin–radixin–moesin
