# Supplementary material for: Promotion of the occurrence of endometrioid carcinoma by S100 calcium binding protein P
Source: BMC Cancer. 2020 Sep 3;20:845. doi: 10.1186/s12885-020-07350-x (PMC7650527; doi:10.1186/s12885-020-07350-x)

1. The result of *S100P* overexpression plasmid sequencing was as follows，which contained Open Reading Frame from 128bp to 415bp. Nonsense mutation was detected at the 225 base of *S100P*（A → C nucleotide change）.

ATGGGCGGTAGGCGTGTACGGTGGGAGGTTTATATAAGCAGAGCTCGTTTAGTGAACCGT

CAGATCGCCTGGAGACGCCATCCACGCTGTTTTGACCTCCATAGAAGATTCTAGAGCTAG

CGAATTCATGACGGAACTAGAGACAGCCATGGGCATGATCATAGACGTCTTTTCCCGATA

TTCGGGCAGCGAGGGCAGCACGCAGACCCTGACCAAGGGGGAGCTCAAGGTGCTGATGGA

GAAGGAGCTACCAGGCTTCCTGCAGAGTGGAAAAGACAAGGATGCCGTGGATAAATTGCT

CAAGGACCTGGACGCCAATGGAGATGCCCAGGTGGACTTCAGTGAGTTCATCGTGTTCGT

GGCTGCAATCACGTCTGCCTGTCACAAGTACTTTGAGAAGGCAGGACTCAAATGATGCCC

TGGATCCGCGGCCGCGAAGGATCTGCGATCGCTCCGGTGCCCGTCAGTGGGCAGAGCGCA

CATCGCCCACAGTCCCCGAGAAGTTGGGGGGAGGGGTCGGCAATTGAACGGGTGCCTAGA

GAAGGTGGCGCGGGGTAAACTGGGAAAGTGATGTCGTGTACTGGCTCCGCCTTTTTCCCG

AGGGTGGGGGAGAACCGTATATAAGTGCAGTAGTCGCCGTGAACGTTCTTTTTCGCAACG

GGTTTGCCGCCAGAACACAGCTGAAGCTTCGAGGGGCTCGCATCTCTCCTTCACGCGCCC

GCCGCCCTACCTGAGGCCGCCATCCACGCCGGTTGAGTCGCGTTCTGCCGCCTCCCGCCT

GTGGTGCCTCCTGAACTGCGTCCGCCGTCTAGGTAAGTTTAAAGCTCAGGTCGAGACCGG

GCCTTTGTCCGGCGCTCCCTTGGAGCCTACCTAGACTCAGCCGGCTCTCCACGCTTTGCC

TGACCCTGCTTGCTCAACTCTACGTCTTTGTTTCGTTTTCTGTTCTGCGCCGTTACAGAT

CCCAAGCTGTGACCGGGCGCCTACGCTAGATGACCGAGTACAAGCCCACGGTGGCGCTCG

CATCGGCGACGACGTCCCCCAGGGGCCGGTACGCACCCTTCGCGACG


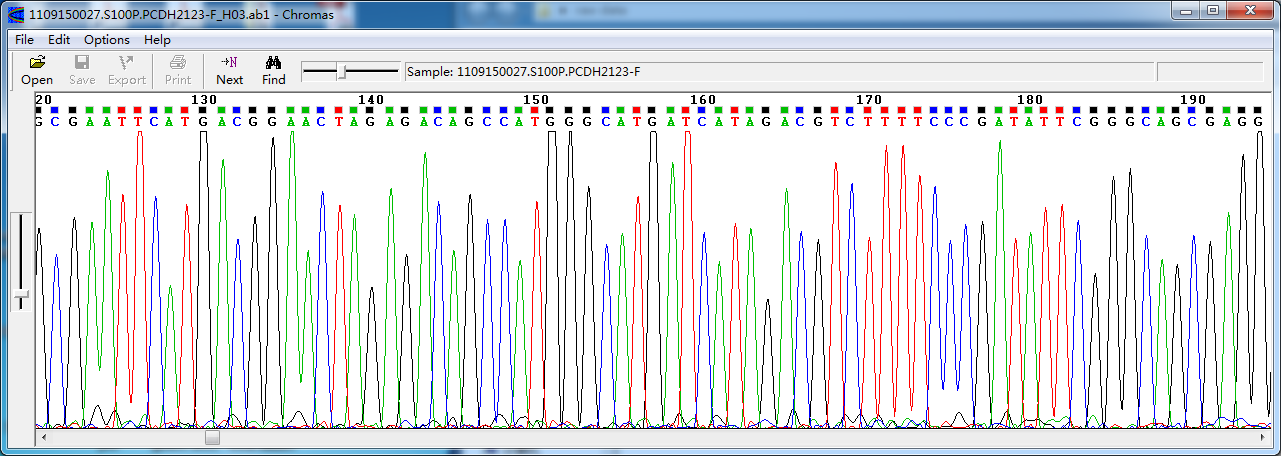


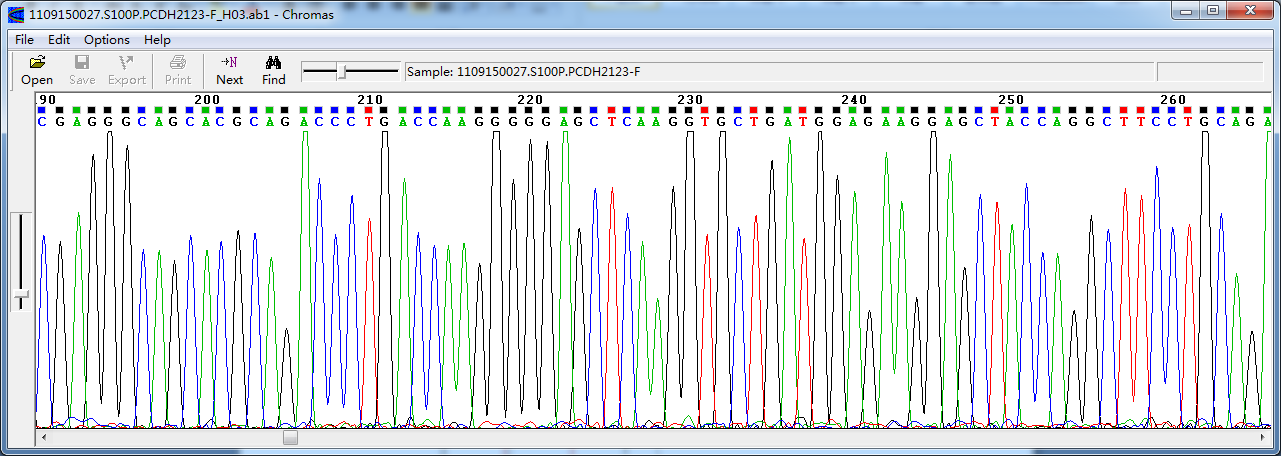


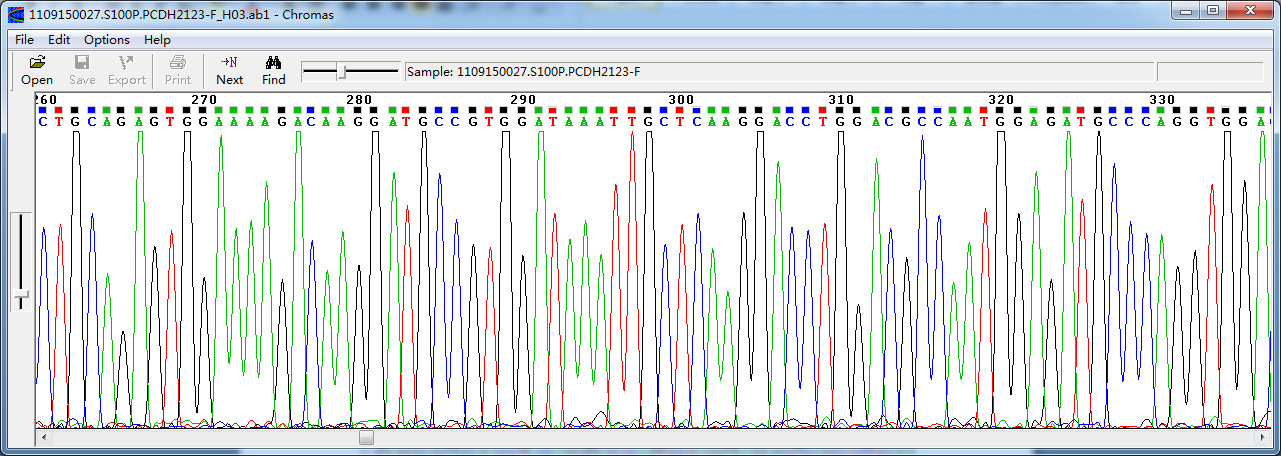


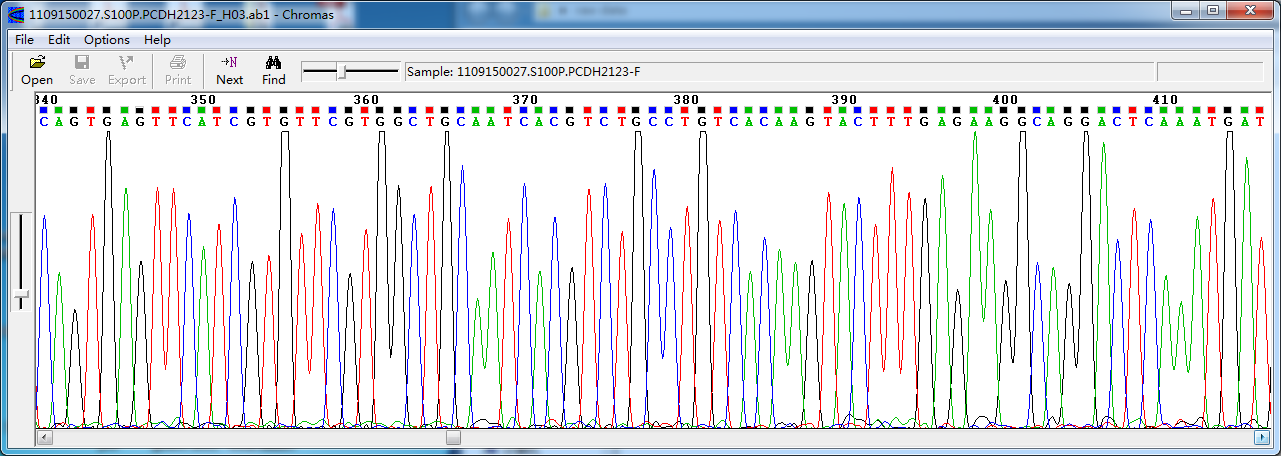


Two sequence alignment were applied.


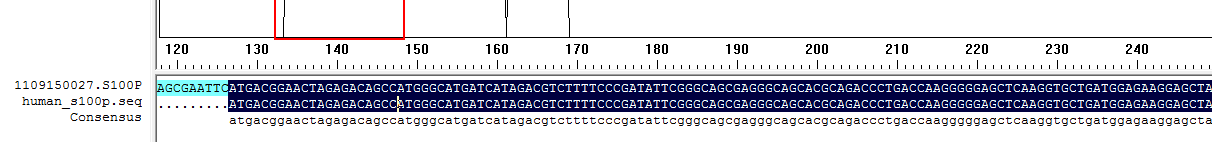


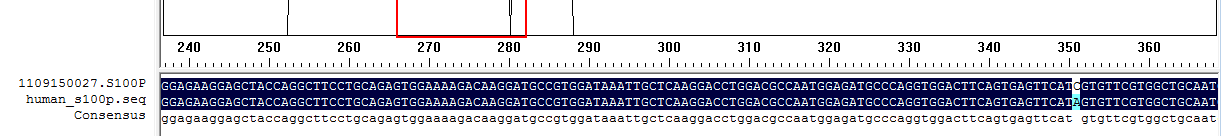

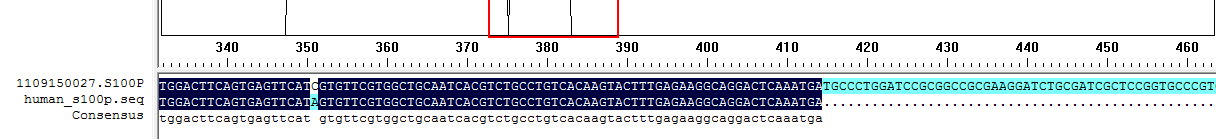


1. The result of *S100P* knocked-down plasmid sequencing was as follows，which contained palindromic structure from 208bp to 265bp.

ACGATACAAGGCTGTTAGAGAGTAATTGGAATTAATTTGACTGTAAACACAAAGATATTA

GTACAAAATACGTGACGTAGAAAGTAATAATTTCTTGGGTAGTTTGCAGTTTTAAAATTA

TGTTTTAAAATGGACTATCATATGCTTACCGTAACTTGAAAGTATTTCGATTTCTTGGCT

TTATATATCTTGTGGAAAGGACGAAACACCGGAAGGATGCCGTGGATAAATTGCTCGAGC

AATTTATCCACGCGCCTTTTTTTGGAATTTCGACCCGGAGACAAATGGCAGTATTCATCC

ACAATTTTAAAAGAAAAGGGGGGATTGGGGGGTACCGTGCAGGGGAAAGAATAGTAGACA

TAAATAGCAACAGACATACAAACTAAAAGAATTACAAAAACAAATTACAAAAAATTCAAA

ATTTTTCGGGGTTATTACAGGGACAGCAGGAGATCCACCTTTGGCCGCGGCTCGAGGGGG

TTGGGGTTGCGCCTTTTTCCAAGGCAGCCCTGGGGTTTGCGCAGGGACGCCGGCTGCCTC

TGGGCGTGGTTCCGGGAAACGGAGCGGCGCCGACCCTGGGTCTCGCACATTCCTTCACGT

CCGTTCGCAGCGTCACCCGGATCTTCGCCGCTACCCTTGTGGGCCCCCCCGGCGACGCTT

CCTGCTCCGCCCCTAAGTCGGGAAGGTTCCTTGCGGTTCGCGGCGTGCCGGACGTGACAA

ACGGAAGCCGCACGTCTCACTAGTACCCTCGCAGACGGACAGCGCCAGGGAGCAATGGCA

GCGCGCCGACCGCGATGGGCTGTGGCCAATAGCGGCTGCTCAGCAGGGCGCGCCGAGAGC

AGCGGCCGGGAAGGGGCGTGCGGGAGCGGGGTGTGGGCGGTAGTGTGGGCCCCTGTTCCT

GCCCGCGCGTGTCCGCATCTGCAAGCTCTGAAGCGCACGTCGGCAGTCGCTCCCTCGTTG

ATCGGATCACGACTCTCTCCCCG


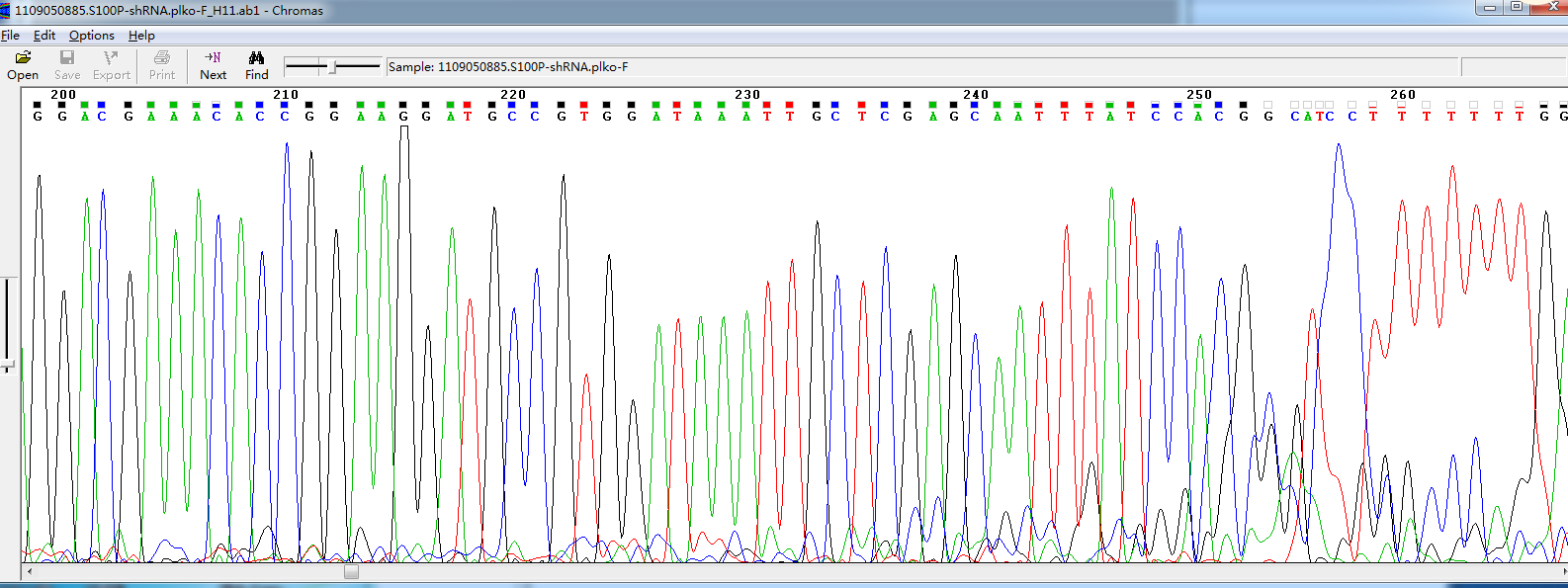


Two sequence alignment were applied.


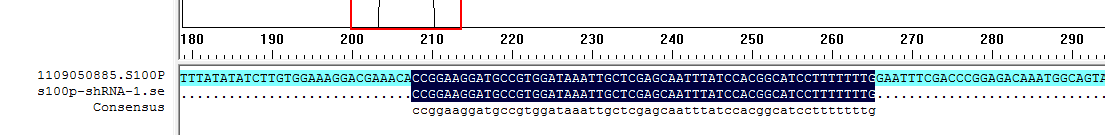

Supplement: Supplementary file 1 — Additional file 1. [file 12885_2020_7350_MOESM1_ESM.docx]
